# A Rare Case of Primary Squamous Cell Carcinoma of the Stomach: A Case Report

**DOI:** 10.7759/cureus.66220

**Published:** 2024-08-05

**Authors:** Abby Kunitsky, Gautam Maddineni, Julia Janecki, Edward Jurkovic, Vladimir Ferrer

**Affiliations:** 1 Gastroenterology, Kansas City University, Kansas City, USA; 2 Internal Medicine, Florida State University College of Medicine, Cape Coral, USA; 3 Internal Medicine, McLaren Macomb, Mount Clemens, USA; 4 Gastroenterology, Lee Health, Fort Myers, USA

**Keywords:** hepatic metastasis, gastric mass, gastric squamous cell carcinoma, chemotherapy, tumor markers, gastric malignancy, squamous cell carcinoma

## Abstract

Pure primary gastric squamous cell carcinoma (PGSCC) is a notably rare gastric malignancy. We present the case of a 51-year-old woman with advanced gastric squamous cell carcinoma characterized by a 7.6 cm necrotic mass invading the proximal stomach, liver metastasis, and lymphadenopathy at diagnosis. Despite the lack of standardized treatment protocols, we review tumor markers and potential management strategies, including surgical and chemotherapeutic interventions. The rarity and aggressive nature of PGSCC necessitates further research to develop effective detection and treatment methods to improve patient prognosis and survival outcomes.

## Introduction

Gastric cancer is one of the most common neoplasms worldwide, yet pure primary gastric squamous cell carcinoma (PGSCC) remains an exceedingly rare malignancy, often with a late presentation, with fewer than 100 cases reported in previous literature [1]. Representing only 0.04-0.4% of all gastric malignancies, this tumor primarily afflicts individuals in their sixth decade, with a male-to-female ratio of 5:1, and is predominantly found in the upper third of the stomach [1-3]. Furthermore, PGSCC cases are deemed more aggressive compared to gastric adenocarcinoma, highlighted by a retrospective review finding the prognosis to be only seven months compared to 11 months for advanced gastric adenocarcinoma [3]. Unfortunately, there is little understanding of the pathogenesis of this rare variant, with neither the National Comprehensive Cancer Network nor the European Society of Medical Oncology having published guidelines [4].

While symptoms may be nonspecific, the disease is often initially discovered via findings on imaging such as computed tomography (CT) or magnetic resonance imaging (MRI), followed by biopsy and histology. Treatment strategy varies and includes surgical intervention with gastrectomy if the disease has not yet metastasized.

In this case, we describe our findings in an adult female patient who was discovered to have a large tumor of the proximal stomach with diffuse liver lesions and multiple enlarged lymph nodes. Once diagnosed, she was transferred to a tertiary care center for further treatment. With limited guidance on management, continued review of published cases may demonstrate a preferred treatment modality.

## Case presentation

**Figure 1:**
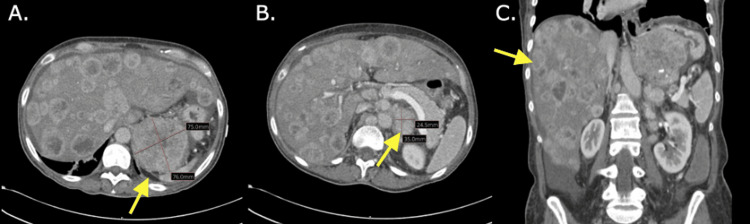
CT of the abdomen/pelvis with contrast. A: Transverse image of the CT showing a large 7.6 cm necrotic mass invading the proximal stomach. B: Transverse image of the CT showing multiple enlarged mesenteric, periaortic, and retroperitoneal lymph nodes, with the largest nodal mass in the left of the aorta measuring roughly 2.4 × 3.5 cm. C: Coronal image of the CT showing innumerable low attenuating liver lesions with peripheral enhancement consistent with diffuse metastatic disease.

A 51-year-old female presented to the emergency department with 10 days of worsening weakness and fatigue. She endorsed five months of progressive shortness of breath, as well as diarrhea and 30 pounds of unintentional weight loss over three months. Physical examination demonstrated tachycardia, moderate epigastric tenderness, and abdominal distention. Labs were notable for leukocytosis (20.3 × 10^3^/µL), thrombocytosis (546 × 10^3^/µL), hyponatremia (120 mmol/L), and elevated alkaline phosphatase (244 U/L), aspartate transaminase (152 U/L), and lactic acid (2 mmol/L). Carcinoembryonic antigen (CEA) and cancer antigen 19-9 (CA 19-9) were elevated at 3.4 ng/mL and 119 U/mL, respectively. Most significantly, a CT of the abdomen/pelvis with contrast revealed a large 7.6 cm necrotic mass invading the proximal stomach, innumerable liver lesions, and multiple enlarged lymph nodes (Figure 1). Gastroenterology, oncology, and nephrology were consulted.

The patient further revealed reduced oral intake due to abdominal bloating, anorexia, and early satiety. She also reported occasional hematochezia, which she attributed to her history of hemorrhoids. She denied any history of esophagogastroduodenoscopy (EGD). Her last colonoscopy was one year prior, with a benign polyp removed. She reported drinking socially and denied currently smoking but did mention smoking cigarettes socially in college. Family history was notable for her mother being diagnosed with rectal, liver, and colon cancer.

**Figure 2:**
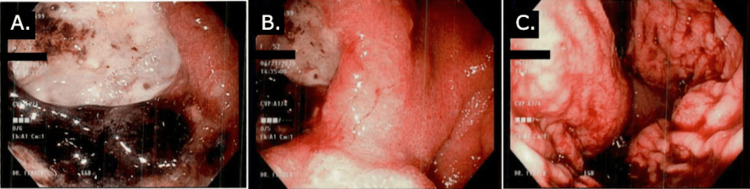
Esophagogastroduodenoscopy pictures. A, B, and C: Multiple angles during esophagogastroduodenoscopy showing a cavitating fundic gastric mass along the lesser curvature with ulceration, heaped-up edges, and necrosis.

**Figure 3:**
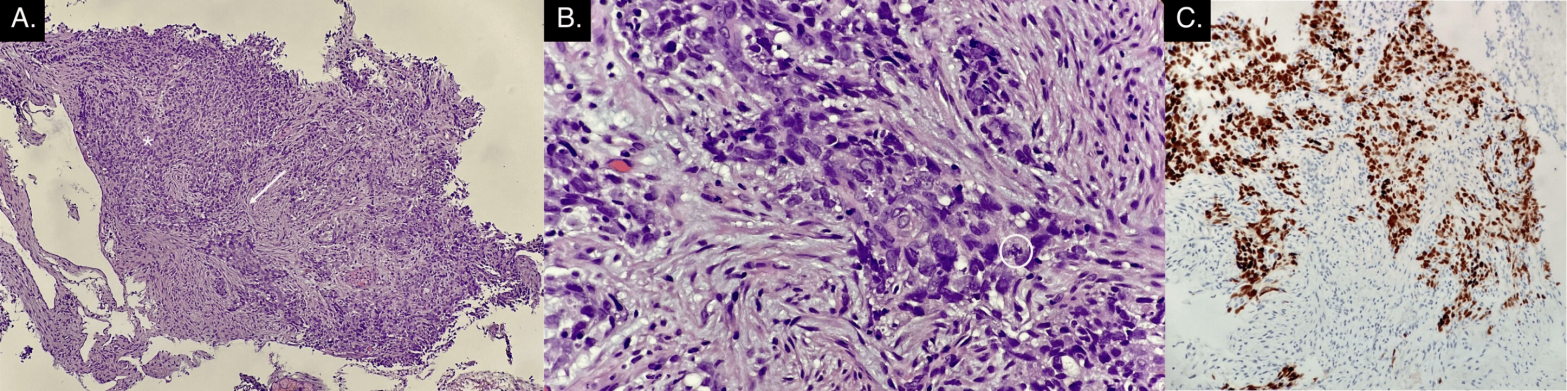
Histopathologic images of gastric mass biopsy. A. Infiltrating malignant cells (*) in a background of desmoplasia (arrow), 4×. B. Poorly differentiated malignant cells (*) with atypical mitosis (circled), 40×. C. Immunohistochemical stain: p40-positive cells, compatible with squamous cell carcinoma, 40×.

EGD revealed a large, nonobstructive cavitating gastric mass occupying the fundus along the lesser curvature with heaped-up edges, extensive central ulceration, and necrosis with friability (Figure 2). Gastric mass biopsy resulted positive for cytokeratin (CK)5, p40, AE1/AE3, Ki-67, and negative for CK7 and synaptophysin, supporting the histologic diagnosis of poorly differentiated squamous cell carcinoma (SCC) with necrosis (Figure 3).

Later in her hospital course, the patient developed anasarca with ascites, requiring three abdominal paracentesis procedures to drain a total of 6.2 L. She was diagnosed with spontaneous bacterial peritonitis; ceftriaxone was started. Ascitic fluid cytology and culture returned negative for malignant ascites. The patient was deemed stable and ultimately transferred to a tertiary hospital for further treatment.

## Discussion

PGSCC has continued as a remarkably rare entity since its description in 1895 [1], posing significant diagnostic and therapeutic challenges due to its aggressive behavior and the lack of standardized management guidelines. While the etiology and pathogenesis are unclear, smoking has been proposed as a potential risk factor, as patients in previously documented case reports had an extensive history of smoking [2,5]. Our patient admitted to only socially smoking during college. Clinical presentations of gastric SCC vary from asymptomatic to nonspecific symptoms such as abdominal pain, nausea/vomiting, weight loss, dysphagia, or gastrointestinal bleeding [1,2,5]. Our patient presented with progressive shortness of breath, diarrhea, and unintentional weight loss over several months.

Diagnosis typically involves CT, EGD with biopsy, and histopathological examination. In this case, CT revealed diffuse liver lesions and lymphadenopathy, EGD showed a large friable gastric mass with extensive ulceration and necrosis, and histopathology demonstrated positive CK5, p40, AE1/AE3, and Ki-67 markers. Tumor markers and molecular studies may facilitate identification for targeted treatment strategies, clinical trial enrollment, or risk classification. Certain PGSCC studies have reported a 99% specificity and 98% sensitivity for p63 and CK5/6 [6]. However, other studies have recommended the inclusion of AE1/AE3, p40, CK7, CK20, TTF1, chromogranin, synaptophysin, CEA, and CA19-9 [1,2].

The most effective therapeutic approach for PGSCC is still being investigated. Surgical resection with or without gastrectomy is the mainstay of treatment, representing a potentially curative option in good surgical candidates with localized disease [1,2,5,6]. Unfortunately, patients are often diagnosed at advanced stages, and survival rates are then expressed in months instead of years [5], thus emphasizing the need for proper detection methods to improve prognosis.

Chemotherapy and radiation for PGSCC remains undetermined and varies based on individual factors and disease characteristics [1,5]. There is inconsistent information regarding the role of neoadjuvant chemotherapy. Previous studies have shown varying success rates with chemotherapy regimens, including 5-fluorouracil-, platin-, and taxane-based regimens such as docetaxel + oxaliplatin/cisplatin + fluorouracil, fluorouracil + oxaliplatin + calcium folinate (FOLFOX), capecitabine + oxaliplatin (XELOX), paclitaxel + carboplatin [2,3,5-7], while others have gone the chemoradiation route [6,8]. A study with 21 PGSCC patients found no significant difference in survival between the surgery group and the surgery plus adjuvant chemotherapy group; however, adjuvant chemotherapy tended to prolong survival time [5]. Many PGSCC studies are only single cases or small case series, highlighting the need for further research on this rare type of gastric carcinoma.

## Conclusions

Pure PGSCC is a rare gastric malignancy with limited treatment guidelines and poor prognosis in advanced stages. In this case, our patient was a nonsmoker with advanced PGSCC characterized by a large necrotic gastric mass and extensive metastasis. The presentation highlights the importance of imaging, procedural, and histopathological evaluation.

Especially given the aggressive nature of PGSCC, there are significant challenges in diagnosing and managing pure PGSCC. There have been a few proposed management strategies in the literature, including surgical resection and various chemotherapy regimens, yet there is a lack of consensus on optimal treatment modalities. Ongoing research and additional case studies on this topic are crucial to establishing evidence-based guidelines and improving patient outcomes.
